# Incidence and risk factors for liver enzymes elevations in highly treatment-experienced patients switching from enfuvirtide to raltegravir: a sub-study of the ANRS-138 EASIER trial

**DOI:** 10.1186/s12981-016-0101-3

**Published:** 2016-04-02

**Authors:** Nathalie de Castro, Joséphine Braun, Isabelle Charreau, Alain Lafeuillade, Jean-Paul Viard, Clotilde Allavena, Jean-Pierre Aboulker, Jean-Michel Molina, The EASIER ANRS 138 study group

**Affiliations:** Department of Infectious Diseases, Assistance Publique Hôpitaux de Paris, Saint-Louis Hospital, University of Paris Diderot Paris 7, 1 avenue Claude Vellefaux, 75010 Paris, France; INSERM SC10-US019, Villejuif, France; Department of Infectiology and Hematology, Font-Pré Hospital, Toulon, France; Department of Infectious Diseases, Hôtel-Dieu Hospital, Paris, France; Department of Infectious Diseases, Hôtel-Dieu Hospital, Nantes, France

**Keywords:** Raltegravir, Tipranavir, Liver enzymes, Transaminases

## Abstract

**Background:**

In the ANRS EASIER trial where treatment-experienced patients switched from enfuvirtide (ENF) to raltegravir (RAL), a high incidence of transaminase elevation was reported in the RAL arm.

**Methods:**

We compared the incidence of emergent liver enzyme elevations (LEE) of grade 2 or more among patients randomized to the maintenance ENF arm or the switch RAL arm up to W24. We also assessed the overall incidence of LEE over the 48-week duration of the trial and baseline risk factors for grade 2 or more alanine aminotransferase (ALT) elevation using univariate and multivariate analyses.

**Results:**

During the first 24 weeks, 6/84 (7.1 %) and 2/85 patients (2.4 %) presented with ALT elevation of grade 2 or more in the RAL and ENF arms, respectively (p = 0.21). Grade 2 or more γGT and ALP elevations were seen in 18 and 11 % (p = 0.35), and 5 and 1 % (p = 0.14) of patients in the RAL and ENF arms, respectively. The 48-week incidence of grade 2 or more LEE was 11.6 per 100-pts-years for ALT, 24.5 per 100-pts-years for γ-GT and 4.5 per 100-pts-years for ALP, respectively. In the multivariate analysis, tipranavir/ritonavir use (OR 3.66; 95 % CI [1.20–11.1], p = 0.022) and elevated ALT at baseline (OR 10.3; 95 % CI [2.67–39.6], p < 10^−3^) were significantly associated with a grade 2 or more ALT elevation during follow-up.

**Conclusion:**

The incidence of LEE was relatively high in these highly treatment-experienced patients switching to a RAL-based regimen. Both tipranavir/ritonavir use and high baseline ALT levels were associated with an increased risk of ALT.

*Trial registration:* ClinicalTrials.gov identifier: NCT00454337

## Background

Liver enzyme elevations (LEE) and in particular alanine aminotransferase (ALT) elevations are frequently reported in patients receiving antiretroviral therapy (ART), although severe ALT elevations (grade 3 or more) are only seen in 2–10 % of patients and do not usually require drug discontinuation [1–3]. Such LEE occur more frequently in patients with underlying liver disease, such as HCV or HBV co-infection and in ART-experienced as compared to ART-naïve patients [1–3]. Different from ritonavir boosted protease inhibitors (PIs/r) and non-nucleoside reverse transcriptase inhibitors (NNRTIs), the liver safety profile of integrase inhibitors and raltegravir (RAL) in particular appears favourable [4]. Indeed, in treatment-experienced patients receiving RAL or placebo in addition to an optimized background regimen (BR), a similar incidence of grade 3–4 ALT elevation was seen at week 48 (4.3 vs 3.4 %) and week 96 (5.4 vs 4.2 %) in the RAL and placebo arms, respectively [5, 6]. As expected, ALT elevations were more frequent in HBV/HCV co-infected patients [4–7]. Also, in another randomized trial among treatment experienced patients, only 2 % of patients who started RAL in addition to the optimized BR experienced a grade 3–4 ALT elevation at week 48, as compared to 3 % for those starting dolutegravir, although 16 % of patients were HBV and/or HCV co-infected [8]. We were therefore surprised observe a higher incidence of LEE in the EASIER ANRS 138 trial in which highly treatment-experienced patients switched from enfuvirtide to RAL in addition to the maintenance of their BR [9]. Indeed, during the first 24 weeks of this trial 21 % of patients in the RAL arm experienced ALT elevations of grade 1 or more. Of note 2 patients experienced a grade 4 ALT elevation requiring raltegravir discontinuation. Both patients were receiving a tipranavir and RAL could be resumed once tipranavir was switched for darunavir [9]. We therefore wished to assess the incidence of LEE in this study and analyze risk factors associated with ALT elevations, including the potential role of tipranavir.

## Methods

### ANRS 138-EASIER trial population

The EASIER ANRS 138 trial was a prospective, multicenter, randomized, 48-week, open-label non-inferiority trial that compared an immediate to a deferred (at week 24) switch from enfuvirtide (ENF) to RAL in highly treatment-experienced HIV-infected patients. A total of 169 patients were enrolled in the study, results have been previously published and showed the non-inferiority of RAL vs ENF [9, 10]. Briefly, eligible patients were integrase inhibitor naïve HIV-1 infected adults with a history of triple class [PI, nucleoside reverse-transcriptase inhibitor (NRTI), and non- nucleoside reverse-transcriptase inhibitor (NNRTI)] failure or intolerance who had achieved virologic suppression with an ENF- based regimen, with plasma HIV-1 RNA levels <400 copies/mL and stable ART for at least 3 months.

### Liver enzymes monitoring

To be enrolled in the study patients had to have ALT levels of less than 2.5 times the upper limit of normal (ULN) (grade 0–1). There were no inclusion criteria relative to alkaline phosphatase (ALP) or γ-glutamyl transferase (γ-GT) levels. LEE was defined by any emergent grade 2, 3 or 4 elevation of either ALT, ALP or γ-GT from baseline to W24 for comparative study (RAL vs ENF) or to W48 for the observational study since all patients in the ENF arm switched to RAL at week 24. Patients were assessed for liver enzyme at baseline, weeks 2, 4, 8, 16, 24 (26 in those switching to RAL at week 24), 28, 32, 40, and 48. Laboratory abnormalities were graded according to the ANRS grading scale for adverse events in adults (http://www.anrs.fr/index.php/content/download/2250/12981/file/ANRS-GradeEI-V6-Fr-2003.pdf). According to this grading scale, a grade 2 elevation of LEE was defined as 2.5–5 times the ULN, a grade 3 as 5–10 times the ULN, and a grade 4 elevation as more than 10 times the ULN.

### Study objectives and statistical analysis

The first objective of this study was to estimate the incidence of emergent grade 2 or more LEE (ALT, ALP, γ-GT) during the study period. The primary comparative intent-to-treat analysis, included all data available up to week 24, and compared the RAL and the ENF arms. A longitudinal analysis was then performed considering all data up to week 48 for all patients. Indeed, from week 24 all patients were receiving a RAL-based regimen. Chi square or Fisher exact tests were used to compare qualitative variables. Kaplan-Meir plots were performed to represent the time to the occurrence of LEE.

The secondary objective of the study was to identify baseline risk factors for emerging ALT elevation of grade 2 or more. A univariate logistic regression model was used, including the following variables: treatment group (RAL vs ENF), age, sex, body mass index (BMI), CD4 cell count, ART duration, history of drug abuse, use of ritonavir boosted tipranavir, ALT elevation at baseline (grade 1 or more), alcohol use, HBV or HCV co-infection, underlying liver disease (liver cirrhosis or steatosis), and use of lipid lowering agents. All variables with a p value below 0.10 were then included in a multivariate model with the addition of alcohol use and underlying liver disease.

Comparisons were made with use of a 2-sided alpha level of 0.05. Statistical analyses were performed with the use of SAS software version 9.2 (SAS Institute Inc, Cary, NC).

## Results

### Characteristics of the patients enrolled in the ANRS-138 EASIER trial

Between June 2007 and September 2007, 170 patients were randomized into the study. One patient withdrew consent before W0 and was therefore excluded from the analysis. The baseline characteristics of the remaining 169 patients were well balanced between both arms (Table 1). Overall, 168 patients (99 %) completed 48 weeks of follow-up. Patients were mostly men (85 %), with a median age of 48 years, and were heavily treatment-experienced with a median duration of ART of 13.6 years and a median duration of ENF of 2.3 years before randomization. Treatment regimen at baseline included ENF (100 %), at least one NRTI (95 %), one or two PIs (99 %), and one NNRTI (8 %). Among the 83 patients of the RAL arm receiving a PI, 37 (44 %) were on TPV and 30 (36 %) were on darunavir (DRV) and among the 85 patients of the ENF arm receiving a PI, 29 (34 %) were on TPV and 34 (40 %) were on darunavir (DRV).

**Table 1:** Baseline characteristics of the patients enrolled in the ANRS 138 EASIER trial

Characteristic	RAL arm	ENF arm
	(n = 84)	(n = 85)
Age (years)		
Median	47.6	48.4
Interquartile range	43.0; 53.7	44.0; 54.3
Male sex, no. (%)	70 (83)	73 (86)
Route of HIV infection, no. (%)		
Sexual	73 (87)	74 (87)
Injection-drug use	5 (6)	5 (6)
Other or unknown	6 (7)	6 (7)
CDC stage, no. (%)		
A	16 (19)	14 (16)
B	26 (31)	24 (28)
C	42 (50)	47 (55)
History of viral hepatitis co-infection		
HBs Ag positive	1 (1)	0 (0)
HCV RNA positive	6 (7)	4 (5)
Cirrhosis	0 (0)	0 (0)
Non alcoolic fatty liver disease	9 (11)	5 (6)
Alcohol consumption		
≥3 times per week	11 (13)	19 (22)
ALAT levels at baseline		
Grade 0	75 (89)	76 (89)
Grade 1	8 (10)	7 (8)
Grade 2	0 (0)	1 (1)
Grade 3	1 (1)	0 (0)
Grade 4	0 (0)	0(0)
Body mass index (BMI)		
Median	22.3	22.6
Interquartile range	19.9; 24.0	20.6; 24.8
Baseline CD4 cells per μL		
Median	410	374
Interquartile range	259; 505	277; 535
Plasma HIV RNA < 50 cp/mL, no. (%)	71 (85)	75 (88)
Duration of prior antiretroviral therapy (years)		
Median	13.7	13.6
Interquartile range	12.1; 15.0	11.7; 15.4
Duration of prior enfuvirtide therapy (years)		
Median	2.5	2.2
Interquartile range	1.6; 3.5	1.4; 3.4
Number of antiretroviral drugs in baseline regimen (including enfuvirtide)		
Median	4	4
Interquartile range	4; 5	4; 5
Antiretroviral drugs in baseline regimen (apart from enfuvirtide)		
Protease inhibitors, no. (%)	83 (99)	85 (100)
Tipranavir/ritonavir	37 (44)	29 (34)
Darunavir/ritonavir	30 (36)	34 (40)
Atazanavir/ritonavir	2 (2)	2 (2)
Lopinavir/ritonavir	9 (11)	15 (18)
Fosamprenavir/ritonavir	11 (13)	11 (13)
NRTIs, no. (%)	79 (94)	81 (95)
Tenofovir	50 (60)	46 (54)
Lamivudine	36 (43)	32 (38)
Emtricitabine	26 (31)	28 (33)
Abacavir	31 (37)	29 (34)
NNRTIs, no. (%)	8 (10)	5 (6)
Nevirapine	5 (6)	1 (1)
Efavirenz	3 (4)	3 (4)
Etravirine	0 (0)	1 (1)

### Incidence of liver enzyme elevations

At baseline, 8–10 % of patients in both arms had grade 1 ALT, and only 2 patients, one in each arm had a grade 2 or 3 ALT elevation (Table 1). Grade 2 or more γ-GT elevations were seen at baseline in 19 and 12 % of patients in the RAL and ENF arms, respectively. Only one patient in the RAL arm had a grade 2 ALP elevation.

During the first 24 weeks of the study, numerically more grade 2 or higher LEE were seen in the RAL arm but the difference did not reach significance (tests adjusted on tipranavir use in BR): 8 grade 2 or more ALT elevations [6 in the RAL arm (7.1 %) and 2 (2.4 %) in the ENF arm, p = 0.21], 24 grade 2 or more γ-GT elevations [15 (18 %) in the RAL arm and 9 (11 %) in the ENF arm, p = 0.35] and 5 grade 2 or more PAL elevations [4 (5 %) in the RAL arm and 1 (1 %) in the ENF arm, p = 0.14].

**Fig. 1:**
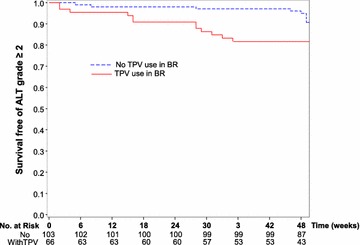
Kaplan-Meier survival probability without grade 2 or more ALT elevation according to the use of tipranavir (TPV) in baseline regimen (BR)

In the observational analysis considering all patients up to week 48, whether or not on RAL, grade 2 or more LEE were distributed as follows: 18 ALT elevations (9 grade 2, 9 grade 3–4) corresponding to an overall incidence of 11.6 events per 100-patients-year (Fig. 1), and to a grade 3–4 incidence of 5.8 events per 100-patients-year, 38 γ-GT elevations (28 grade 2, 10 grade 3–4) corresponding to an incidence of 24.5 events per 100 pts-year and 7 PAL (5 grade 2, 2 grade 3–4) corresponding to an incidence of 4.5 events per 100 pts-year. Among the 9 patients with grade 3–4 ALT elevations, tipranavir was replaced by either darunavir (n = 8) or lopinavir (n = 1) and RAL could be resumed in the 3 patients (all in the RAL arm) in whom it was temporarily discontinued. Of note, among the 11 patients (6.5 %) who were HBV (HBs Ag positive) or HCV (HCV RNA positive) co-infected in this trial, only one patient with HCV co-infection experienced a grade 2 ALT elevation.

### Risk factors for ALT elevation

In order to identify baseline risk factors of emergent grade 2 or more ALT elevation we first performed a univariate logistic regression model. Among all baseline variables tested, two were significantly associated with emergent grade 2 or more ALT elevation (Table 2): elevated ALT (grade 1 or more) with an OR of 8.78 (95 % CI [2.80–27.6], p < 10^−3^) and tipranavir use in BR with an OR of 3.56 (95 % CI [1.26–10.0], p = 0.016). In the multivariate analysis, these two factors remained significantly associated with the occurrence of emergent ALT elevation of grade 2 or more (Table 2).

**Table 2:** Univariate and multivariate analysis of baseline risk factors associated with grade 2 or more ALT elevation

	Odds ratio	95 % CI	p value
Univariate analysis			
Raltegravir vs enfuvirtide	1.66	(0.61–4.51)	0.322
Age (↑ 10 years)	1.12	(0.62–2.05)	0.702
Sex (female/male)	2.36	(0.76–7.31)	0.136
Body mass index (BMI) (↑ 5)	0.73	(0.31–1.70)	0.469
CD4 cell count (↑ 100 cells/µL)	1.08	(0.87–1.34)	0.502
ART duration (↑ 1 year)	0.86	(0.70–1.05)	0.149
History of drug abuse	0.92	(0.11–7.73)	0.940
Use of boosted tipranavir at baseline	3.56	(1.26–10.0)	0.016
ALT elevation (grade 1 or more)	8.78	(2.80–27.6)	<10^−3^
Alcohol use (>2 times/week)	0.54	(0.12–2.51)	0.435
Hepatitis B or C co-infection	0.82	(0.10–6.84)	0.857
Liver disease at baseline (steatosis/cirrhosis)	2.53	(0.63–10.1)	0.189
Use of lipid lowering agents	2.06	(0.76–5.57)	0.156
Multivariate analysis			
Use of boosted tipranavir	3.66	(1.20–11.1)	0.022
ALT elevation (grade 1 or more)	10.3	(2.67–39.6)	<10^−3^
Alcohol use (>2 times/week)	0.39	(0.07–2.16)	0.281
Liver disease at baseline (steatosis/cirrhosis)	0.89	(0.16–5.01)	0.899

## Discussion

In this trial among treatment-experienced patients virologically suppressed under an ENF-based regimen combining ritonavir boosted PIs and NRTIs, the switch from ENF to RAL to avoid parenteral injections of ENF, was not associated with a significant increase in LEE. Indeed, during the first 24 weeks of this randomized trial, only 6/84 (7.1 %) and 2/85 patients (2.4 %) presented with ALT elevations of grade 2 or more in the RAL and ENF arms, respectively (p = 0.21). Also, the frequencies of grade 2 or more ALT and γ-GT elevations were not significantly higher in the RAL as compared to the ENF arm. However, the 48-week incidence of grade 2 or more ALT elevation was 11.6 per 100-pts-years in this study, and that of grade 3 or 4 ALT elevation was 5.8 per 100-pts-years, an incidence somewhat higher than previously reported at W48 in treatment-experienced patients receiving RAL-based regimens: 4.3 % in the BENCHMRK study and 2 % in the SAILING study [5–8].

To better understand this observation we performed a multivariate analysis, to identify the baseline risk factors associated with a new grade 2 or more ALT elevation during the 48-week duration of the study (Table 2). Two baseline variables were significantly associated with such an ALT elevation: tipranavir/ritonavir use in the BR (OR 3.66; 95 % CI [1.20–11.1], p = 0.022) and elevated ALT at baseline (grade 1 and more) (OR 10.3; 95 % CI [2.67–39.6], p < 10^−3^) whereas the use of RAL was not significantly associated to such an emergent increase in ALT levels.

These results might therefore explain the relatively high incidence of LEE in this trial. Indeed tipranavir use has been previously associated with a high incidence of ALT elevations in particular among HCV or HBV co-infected patients, possibly also because of the double dose of ritonavir (200 mg bid) used to boost tipranavir as compared to only 100 mg bid for most of the other PIs (darunavir and lopinavir) [11]. Also, in pooled safety analyses from phase II and III tipranavir/ritonavir development trials, during the 96 weeks follow-up of 1299 patients receiving tipranavir/ritonavir, 11 % of patients experienced a grade 3–4 ALT elevation [12]. In our trial a sizeable proportion of patients (66/168; 39 %) were receiving tipranavir at baseline, slightly more in the RAL (44 %) than in the ENF (34 %) arm. In other trials of treatment experienced patients starting RAL in combination with a boosted PI, lower proportion of patients were receiving tipranavir/ritonavir, only 22 % of patients in the Benchmrk trial, less than 20 % in the Sailing trial and 0 % in the ANRS TRIO trial, and this may account for the differences seen in the incidence of LEE elevations across these trials [5–7, 13].

Close monitoring of LEE and ALT elevation in particular is recommended when patients are receiving ritonavir boosted-tipranavir, and in case of significant ALT elevation a switch to a more liver-friendly PIs such as darunavir or lopinavir could be tested. Indeed, in the 9 patients who discontinued RAL because of grade 3–4 ALT elevations during our study, all under a ritonavir/tipranavir-based regimen, RAL could be resumed in combination with a new boosted PI. RAL would also be unlikely to increase tipranavir liver toxicity since a previous pharmacokinetic sub-study of the ANRS EASIER trial showed that the switch from ENF to RAL lowered plasma concentrations of tipranavir [14]. Elevated ALT at baseline was also identified as a risk factor for the occurrence of a new ALT elevation during follow-up as previously reported [15]. Close monitoring of ALT levels should also be performed in such patients.

## Conclusion

Among highly treatment experienced patients RAL is not associated per se with an increase in LEE but liver enzymes and ALT should be carefully monitored in patients receiving a tipranavir-based regimen or with elevated baseline ALT levels.
